# Music synchronizes brainwaves across listeners with strong effects of repetition, familiarity and training

**DOI:** 10.1038/s41598-019-40254-w

**Published:** 2019-03-05

**Authors:** Jens Madsen, Elizabeth Hellmuth Margulis, Rhimmon Simchy-Gross, Lucas C. Parra

**Affiliations:** 1 Department of Biomedical Engineering, City College of New York, New York, USA; 2 Department of Music, University of Arkansas, Fayetteville, Arkansas USA; 3 Department of Psychological Science, University of Arkansas, Fayetteville, Arkansas USA

## Abstract

Music tends to be highly repetitive, both in terms of musical structure and in terms of listening behavior, yet little is known about how engagement changes with repeated exposure. Here we postulate that engagement with music affects the inter-subject correlation of brain responses during listening. We predict that repeated exposure to music will affect engagement and thus inter-subject correlation. Across repeated exposures to instrumental music, inter-subject correlation decreased for music written in a familiar style. Participants with formal musical training showed more inter-subject correlation, and sustained it across exposures to music in an unfamiliar style. This distinguishes music from other domains, where repetition has consistently been shown to decrease inter-subject correlation. Overall, the study suggests that listener engagement tends to decrease across repeated exposures of familiar music, but that unfamiliar musical styles can sustain an audience’s interest, in particular in individuals with some musical training. Future work needs to validate the link proposed here between music engagement and inter-subject correlation of brain responses during listening.

## Introduction

One of the most basic and universal responses a person can have to music is engagement. When listeners are engaged with music, they follow the sounds closely, connecting in an affective, invested way to what they hear. Despite the importance of this engagement, it has been difficult to study given the limits of self-report and physiological measures, especially in a domain like classical music where listeners are accustomed to sitting quietly without providing overt evidence of their internal experience.

A domain with a similar problem is film, where viewers might sustain powerfully involving experiences, but remain motionless and silent in their seat. Dmochowski *et al*. showed that inter-subject correlation in neural activity as measured by EEG corresponds with arousing moments–such as a close-up of a weapon–within rich cinematic narratives, but not within casual footage of everyday life^1^. When participants engage with a film–where ‘engagement’ is defined behaviorally as a commitment to watch the film^2^–their neural responses often track occurrences in predictable ways that are shared from viewer to viewer. Thus, previous work has shown that the level of inter-subject correlation is correlated with audience retention assessed behaviorally. This work validated ISC as a neural predictor of how well a film can capture its audience, in a fairly literal sense^2^. Similarly, synchrony between classroom students’ brains predicts classroom engagement^3^. Thus, inter-subject correlation of neural responses is also potentially well-suited for measuring musical engagement^4^, despite that the relationship between engagement and ISC is not perfect (a period with low ISC, for example, might suggest either that listeners are unengaged or that they are engaged by diverse parts of the stimulus). By exposing people to excerpts of instrumental music and tracking the inter-subject correlation of their neural response, this paper aims to assess engagement implicitly. We postulate that inter-subject correlation is also a good predictor for musical engagement. While we will not test this explicitly, we will perform a number of manipulations that are expected to affect music engagement and we will track how these affect inter-subject correlation of the EEG.

One characteristic of music that distinguishes it from film is the prevalence of repetition–not only do individual songs feature copious repetition (a chorus that recurs again and again, for example), but also listeners tend to play and replay their favorite tracks^5^. When watching film, inter-subject correlation decreases when participants watch a clip the second time, suggesting that the film has become less engaging or the audience’s attention has started to wander^6^. Because repetition plays such a significant role in music, this paper also aims to address whether repeated exposures influence engagement differently for musical examples.

A large body of research in psychoaesthetics traces the inverted-U shaped curve in “hedonic value” (which might be thought about as some kind of composite of enjoyment, interest, and attentiveness) across multiple exposures to a particular stimulus–especially a piece of music^7^. As listeners encounter a piece again and again, they tend to enjoy it more and more, until a threshold beyond which liking diminishes with further repetitions^8^. This curve is reliably modulated by stimulus complexity. For an intricate, challenging stimulus, it can take more repetitions to reach the peak of the curve, whereas for a simple one, the plateau can arrive more quickly^9^. Stimulus complexity is not determined purely by acoustic properties; listener experience also plays a role. For a listener without experience in a given style, an excerpt might seem quite complex, but to a listener well versed in the style–able to chunk and process patterns with ease–the same excerpt might seem simple. In our study, we theorized that people with formal musical training would likely have more experience with the type of instrumental excerpts used as stimuli, rendering them comparatively less complex. They may also discern more structure in the music and find it comparatively more engaging^10^.

In order to assess engagement across repeated exposures, this paper uses excerpts of instrumental classical music. Since good theories of moment-to-moment engagement exist for this repertoire, this choice of stimuli allows an assessment of the degree to which inter-subject correlation serves as a useful measure. Moreover, since the music involves only instruments rather than singing, there is no linguistic content to influence the responses. Participants listened multiple times to each excerpt in immediate succession. Half of the excerpts were composed in the common-practice style ubiquitous in concert halls and media soundtracks. Half of the excerpts used musical materials that are less prevalent. When listeners have experience with a particular style, they can parse the music more easily, rendering it simpler. When they lack such experience, however, the music can seem more complex and difficult^11^. In keeping with previous research on the inverted-U response, engagement (and thus inter-subject correlation) might increase across repetitions of music in an unfamiliar style, and decrease across repetitions of music in a familiar style, according to where the piece starts on this inverted-U.

## Method

### Participants

A total of 40 participants took part in the study, each in one of three experiments. Experiments 2 and 3 were designed to replicate and extend the findings from Experiment 1. In the first experiment a cohort of 20 participants took part (11 female and 8 male, age 18–22 M = 19, SD = 1.15). All participants were undergraduate students at the University of Arkansas, and none were music majors.

In the second and third experiment a cohort of 20 participants took part (8 female and 12 male, age 18–34 M = 23.65, SD = 4.67). The second experiment was a replication of the first experiment using the original stimuli (N = 8 pieces). The third experiment tested additional stimuli (N = 12 pieces). Among these 20, 10 participants had received musical training for more than 1 year but none were music majors. Participants in the second experiment were sampled from a culturally diverse group of undergraduate and graduate students at The City College of New York.

The experiment protocol was approved by the Institutional Review Boards of the University of Arkansas and the City University of New York. All methods were carried out in accordance with relevant guidelines and regulations. Written informed consent was obtained from all subjects.

### Materials

Stimuli were excerpts of instrumental classical music composed in a familiar or unfamiliar style, extracted from commercially available recordings (see Table 1). Pilot work ensured that the individual excerpts were not broadly known, and that the style for each excerpt was perceived as broadly familiar or unfamiliar. For Experiment 1 and 2, excerpts were approximately 90 s in duration (see Table for exact duration). For Experiment 3, excerpts were 60 s long. The shorter duration in Experiment 3 allowed for the presentation of more stimuli in the experiment (12 instead of 8).

**Table 1:** Overview of stimuli. Composer, title, duration, style of the stimuli and the slope of ISC (multiplied with 1000 for readability) computed across repetition for the trained participants in experiment 2 and 3.

Experiment	Composer	Title	Duration	Familiarity rating	Style	ISC slope
3	Gioachino Rossini	La Gazza Ladra: Overture	1:00	6	Familiar	−12.9
1&2	Franz Liszt	A Faust Symphony, S. 108: 3. Mephistopheles	1:33	6	Familiar	−6.4
1&2	Felix Mendelssohn	String Quartet No. 3 in D Major, Op. 44 No. 1, I. Molto allegro vivace	1:46	6	Familiar	−5.0
3	Arnold Schoenberg	Five Orchestral Pieces Op. 16 I. Vorgefühle	1:00	2	Unfamiliar	−4.2
1&2	Franz Schubert	Piano Sonata No. 20 in A Major, D. 959: 3. Scherzo (Allegro vivace)	1:39	7	Familiar	−4.1
1&2	Anton Webern	Symphony, op. 21: II. Variationen	1:46	4.5	Unfamiliar	−2.7
3	William Grant Still	Symphony No. 1, Movement 1	1:00	5.5	Familiar	−1.9
3	Ludwig van Beethoven	Egmont Overture, Op. 84	1:00	5	Familiar	−1.6
1&2	Igor Stravinsky	Piano Sonata (1924), Movement 1	1:41	5	Unfamiliar	−1.5
3	Camille Saint-Saëns	Cello Concerto No. 1 in A Minor, Op 33 I. Tempo 1: Allegro non troppo	1:00	6	Familiar	−1.5
3	Felix Mendelssohn	Symphony No. 1 in C Minor, Op. 11, IV: Allegro con fuoco	1:00	6	Familiar	−0.7
1&2	Wolfgang Amadeus Mozart	Symphony No. 24 in B Flat, K. 182: 1. Allegro Spiritoso	1:41	6	Familiar	0.7
3	Béla Bartók	Violin Concerto No 2. Sz. 112, I. Allegro non troppo	1:00	3	Unfamiliar	1.0
3	Georges Bizet	Symphony In C, I. Allegro vivo	1:00	5.5	Familiar	1.1
3	Philip Glass	String Quartet No. 5 - Part 3	1:00	5	Unfamiliar	1.2
1&2	György Ligeti	String Quartet No. 1, “Metamorphoses nocturnes”	1:33	5	Unfamiliar	2.3
3	Yu Xunfa	Harvest	1:00	5	Unfamiliar	3.4
3	Silvestre Revueltas	Homenaje a Garcia Lorca: 1. Baiile	1:00	3.5	Unfamiliar	3.9
1&2	Arnold Schoenberg	5 Orchestral Pieces, Op. 16 No. 5 Das obligate Resitativ	1:47	2	Unfamiliar	4.8
3	Thomas Adès	These Premises Are Alarmed	1:00	3.5	Unfamiliar	5.2

A negative slope indicates there is a decrease in ISC and a positive slope indicates there is an increase in ISC as a function of repeated exposure to music. Since ISC functions as a measure of engagement, slope across repeated listenings tracks the effect of repetition on listener engagement.

For the second and third experiment, the sound level of each stimulus was roughly normalized for volume. Table 1 lists details for all the stimuli used in the experiment. Additionally, to ensure that the physical properties of each musical piece was not the driving factor behind ISC, but only engagement, the spectral dynamics was computed as a measure of the dynamics in each musical piece (see detailed description below). Each piece for Experiment 3 was selected from a pool of 20 pieces, so that the combined stimuli for Experiment 2 and 3 had no significant difference in terms of spectral dynamic between the familiar and unfamiliar conditions.

### Procedure

In Experiments 1 and 2, each participant listened to 8 excerpts three times each. The excerpt order was randomized for each participant, but excerpt presentation was blocked, so that they heard each piece three times in a row before proceeding to the next one. Each participant was asked to listen as attentively as possible, while looking at a fixation cross on a screen to minimize eye movements and ensuring a maximum of alertness with eyes open. In the third experiment each participant listened to 12 musical excerpts, again each repeated 3 times in immediate succession. In Experiment 2 and 3, after each participant listened to the block-randomized stimuli, they listened to the pieces once more in an distract condition. In this condition each stimulus was presented a single time, while participants counted backwards, from a randomly chosen prime number between 800 and 1000, in decrements of 7. These task instructions aimed to distract the subjects from the stimulus without requiring overt responses^6^. After the counting condition, participants filled out questionnaires regarding their musical background and experience with the stimuli in the study (familiarity and preference). For these questions, all stimuli and response options were presented on the same screen to encourage relative ratings.

### Preprocessing of EEG data

In the first experiment, the EEG was recorded with a Brain Amp DC amplifier at a sampling frequency of 1000 Hz. Participants were fitted with a standard, 60-electrode cap following the international 10/10 system referenced to FCz (location file was not available). The electrooculogram (EOG) was recorded with four auxiliary electrodes, two electrodes positioned adjacent to the outer canthi of the left and right eye, and two centered above and below the left eye. For segmentation of the musical stimuli, onset triggers were used. In the second and third experiment, the EEG was recorded with a BioSemi Active Two system at a sampling frequency of 512 Hz. Participants were fitted with a standard, 64-electrode cap following the international 10/10 system. The electrooculogram (EOG) was also recorded with six auxiliary electrodes (one located dorsally, ventrally, and laterally to each eye). For segmentation of the musical stimuli, tone bursts were embedded before and after the musical stimuli and a Cedrus StimTracker was used to identify onset and offset of each stimuli.

The EEG and EOG data were first analog band-pass filtered between 0.016 to 250 Hz. The signal was then high-pass filtered (0.5 Hz cutoff) notch filtered at 60 Hz. Robust PCA^12^ was used to remove artifacts and outliers, and subsequently the signal was low-pass filtered (64 Hz cutoff) and down-sampled to 128 Hz. Segments of the EEG and EOG were extracted corresponding to each musical piece and their repeats. Electrode channels with high variance were manually identified and replaced with interpolated channels. The interpolation was performed using the 3D cartesian coordinates from the electrode cap projected onto a plane. An interpolation method was used to recreate each channel, derived from all surrounding “good” electrodes. The EOG channels were used to remove eye-movement artifacts by linearly regressing them from the EEG channels, i.e. least-squares noise cancellation^13^. In each EEG channel, outlier samples were identified (values exceeding 4 times the distance between the 25th and the 75th quartile of the median-centered signal) and samples 40 ms before and after such outliers were replaced with zero valued samples.

### Inter-Subject Correlation of EEG

**Figure 1:**
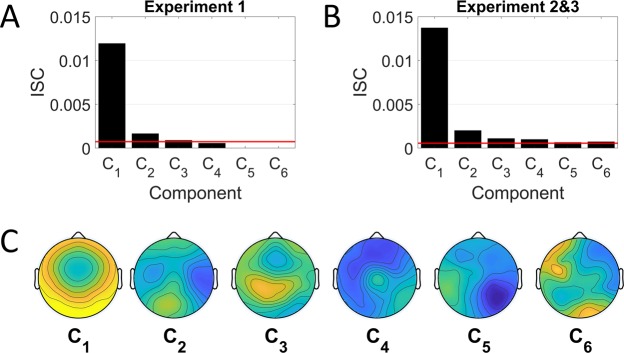
The ISC values (cross-validated) for each component for the concatenated test for Experiment 1 (**A**) and Experiment 2&3 (**B**). The red line indicates the single largest ISC value for all circular shifted test sets. All components larger than this cutoff are statistically significant. (**C**) Scalp topographies corresponding to the significant components found for Experiment 2 and 3.

Inter-subject correlation (ISC) of the EEG evoked activity was computed using correlated component analysis^14^. The method finds a model that linearly combines electrodes that capture EEG activity most correlated between participants. The model is comprised of several projection vectors that linearly combines electrodes, on which the data is projected (components), that capture significant correlation between subject. Correlated component analysis is conceptually the same as canonical correlation analysis (CCA) or multi-set CCA, but differs in that the same projection vectors are used for all subjects. In the analysis we induced EEG recordings during listening to music for all participants, ensuring that the component projections are common to all participants (see the resulting “forward model” topography^14^ of these components in Fig. 1B). The ISC of each component is obtained by computing the correlation coefficients of the projected EEG time-course between each participant and all other participants (see the resulting ISC values and the significance threshold in Fig. 1A). The ISC calculation is identical to previously published implementations and can be reproduced with code available at http://www.parralab.org/isc/.

Statistically significant components are found on test-set performance. We train the model for all pieces, leaving one piece out, and measure ISC on the left-out music piece. This is repeated for all pieces to obtain test set ISC on all pieces. Significance of the resulting ISC values are computed with circular shifted statistics. Specifically, the waveforms are circularly shifted in time at random for each subject, and ISC recomputed in this random-shift data. This is repeated 100 times, and the largest (shuffle) ISC value among all components is used as significance threshold (red line in Fig. 1A). For Experiment 1 we find 2 significant components and for Experiment 2 and 3 we find 3 significant components (consistent with our previous work on film and speech^6,15,16^. For the combined analysis of Experiment 2 and 3 we find 6 significant components, consistent with the increased power due to the larger dataset.

Following previous research, ISC is calculated by using the sum of all significant components of the EEG that capture correlated responses between subjects (sum of values above the red line in Fig. 1A). For the investigation into the change in ISC as a function of repeat, a separate component model was trained for each Experiment 1, 2 and 3. For the analysis of the effect of musical training, a single component model was trained combining Experiment 2 and 3. For the verification of neural engagement analysis, a single components model was trained combining the attend and distract conditions.

### Spectral dynamic computation

We expect that more dynamic stimuli will cause stronger EEG evoked responses and lead to higher ISC^16^. Experiment 2 and 3 controlled for this aspect by computing a measure of spectral dynamics (conventionally called spectral flux), and selecting stimuli with matched spectral dynamics for the familiar and unfamiliar categories. The spectral dynamic was computed using the Gammatonegram^17^, with a 128 filter gammatone filter bank using center frequencies determined by the critical band. The signal was divided into 20 ms Hanning windowed frames with a 10 ms overlap. The power of each frame for each frequency bin was computed and the spectral dynamic was computed by taking the Euclidean distance between each successive frame. Following previous work^16^ linking ISC of EEG evoked responses to stimulus dynamics, we aggregated these spectral dynamic (distance) measures as follows: We applied a 95th percentile filter with a window size of 1 second followed by a median filter with a 5 second window. The goal of this was to obtain the largest changes in spectra which were expected to robustly drive the EEG evoked responses^16^. The resulting set of spectral distance values (spectral dynamic) were combined for all music pieces in the familiar or unfamiliar categories. Pieces were selected for each category so that these pooled spectral dynamic values were not statistically different when a t-test was applied between familiarity conditions (the final selection was *p* = 0.8).

All data used in this study is available upon request from the authors.

## Results

Experiment 1 aimed to test how brain activity changes when a musical excerpt is repeated several times in immediate succession, and how this interacts with the familiarity of the pieces. Participants (N = 20) passively listened to classical instrumental music composed in a familiar or unfamiliar style. Excerpts in a familiar style use sounds and patterns that tend to be more common in the everyday lives of the study’s participants; excerpts in an unfamiliar style use sounds and patterns that pilot work suggested are comparatively rarer (see Table 1). Pieces were presented in a randomized block design, each block consisting of a single piece repeated three times. Neural activity was recorded during listening with a 64 channel EEG system. The ISC was computed for each musical piece for all participants, by measuring the correlation of EEG evoked activity of each individual to the activity in all other participants in the group (see Methods). The ISC across all participants can be found in Fig. 1 for different components of the EEG (each component is a linear combination of EEG sensors that captures correlated evoked activity between participants).

### ISC drops with repetition of familiar music but not unfamiliar music

**Figure 2:**
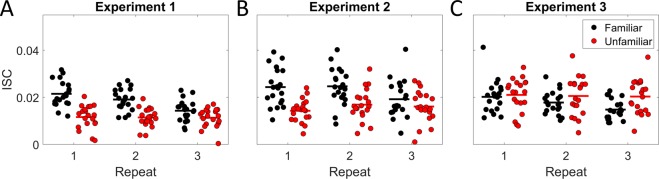
ISC drops with repeated exposure of music in a familiar style. ISC values for each participant (each circle is a participant), averaged across stimuli in the familiar (black) and unfamiliar style categories (red). Each piece is repeated three times before the next piece is presented. (**A**) In the first experiment ISC drops with repeated exposures, but not for excerpts in an unfamiliar style. (**B**) Experiment 2 replicates Experiment 1 on a different participant cohort and (**C**) Experiment 3 replicates findings in Experiment 1 and 2 on different stimuli.

Figure 2A shows the resulting ISC values for each participant in Experiment 1. ISC values are higher for pieces in a familiar style as compared to unfamiliar. For the excerpts in a familiar style, ISC drops on the second and third presentation, but not for excerpts in an unfamiliar style, where ISC seems to be sustained across repeats. This observation was tested using a two-way repeated-measures ANOVA (repeated measures on the same participants), testing for effects of repeating the stimulus and the familiarity of the style. This resulted in a main effect of repetition (*F*(2, 95) = 12.52, *p* = 1.50e-05), a main effect of familiarity (*F*(1, 95) = 121.46, *p* = 1.10e-18) as well as an interaction between repetition and familiarity (*F*(2, 95) = 10.63, *p* = 6.80e-05). This confirms our main hypothesis that ISC drops with repeated exposure to music in a familiar style, but not for music in an unfamiliar style. This is consistent with our interpretation of ISC as a correlate of music engagement—familiar music starts off as more engaging, but might quickly become boring, whereas unfamiliar music might remain interesting across repetitions.

Among the participants there was significant variability in ISC (*F*(19, 460) = 3.63, *p* = 5.18e-07). Given this variability we wondered whether the results were dominated by the specific cohort of participants. We thus repeated Experiment 2 on a new cohort recruited at a different location with a more diverse demographic (N = 20). The findings from the first experiment were fully replicated as seen on Fig. 2B. Performing a two-way repeated measure ANOVA on ISC for the second cohort showed again a main effect of repetition (*F*(1, 97) = 6.15, *p* = 1.49e-02), familiarity (*F*(1, 97) = 30.62, *p* = 2.67e-07) and an interaction between repetition and familiarity (*F*(1, 97) = 7.94, *p* = 5.87e-03). These results were obtained by averaging over 4 musical pieces in each category (familiar/unfamiliar). Among the pieces there was also significant variability in ISC for Experiment 1 (*F*(7, 472) = 17.88, *p* = 4.26e-21) and Experiment 2 (*F*(7, 625) = 16.77, *p* = 2.36e-20). Given the small sample size of musical pieces and the substantial variability, we wanted to test if these results would replicate on a different selection of musical pieces. In Experiment 3, we chose a new set of 12 pieces with more varied excerpts of music. In our selection, we controlled for the physical properties in the music by balancing the spectral dynamics of pieces between the two familiarity conditions (see Methods). Using the same participant cohort as Experiment 2, we again found a significant main effect of repeat (*F*(1, 97) = 9.48, *p* = 2.70e-03). Although there was no main effect of familiarity (*F*(1, 97) = 0.88, *p* = 0.35), there was an interaction between repetition and familiarity (*F*(1, 97) = 3.90, *p* = 0.05). This replicates the results of Experiment 1 and 2, with ISC sustaining across repeated exposures for unfamiliar but not for familiar styles of music.

### ISC is higher in participants with musical training

**Figure 3:**
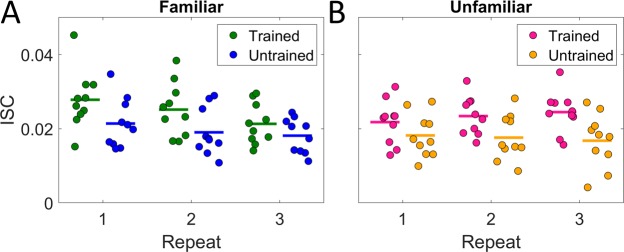
ISC with repeated exposure of music for musically trained and untrained participants. Average ISC values across stimuli for each participants (each circle represents a participant) (**A**) Average over 10 familiar pieces. (**B**) Average over 10 unfamiliar pieces. There is a clear difference in ISC between participants with and without training (at least one year of instruction in a musical instrument). Data combines Experiment 2 and 3.

Musical training might dispose a person to engage more readily with unfamiliar music, since trained listeners likely have more experience grappling with and learning new musical materials. Experiment 2 and 3 recruited 10 participants with some training on a musical instrument (more than 1 year, self-reported), and 10 without such training. To investigate the effect of this training on the ISC values we separated the analysis by training of participants, but averaged all 10 pieces in each category (familiar/unfamiliar), combining Experiment 2 and 3. The resulting ISC values for each participant can be seen on Fig. 3A for familiar and Fig. 3B for unfamiliar music styles. The overall difference in ISC across all repeats for participants with musical training is apparent for both the familiar and unfamiliar styles. For music in familiar styles, ISC decreases across repetition for the trained participants, but not for untrained participants (Fig. 3A). These finding were verified using a 3-way repeated measures ANOVA, with repeated measures over participants, and fixed factors of repeat, familiarity and musical training. As before, we find a significant main effect of repetition (*F(*1, 97) = 11.63, *p* = 9.47e-04), familiarity (*F*(1, 97) = 12.23, *p* = 7.11e-04) and an interaction between the two (*F*(1, 97) = 8.20, *p* = 5.14e-03). Importantly, now also we find a main effect of musical training (*F*(1, 97) = 6.62, *p* = 1.91e-02).

### The effect of repetition on ISC is modulated by musical training

**Figure 4:**
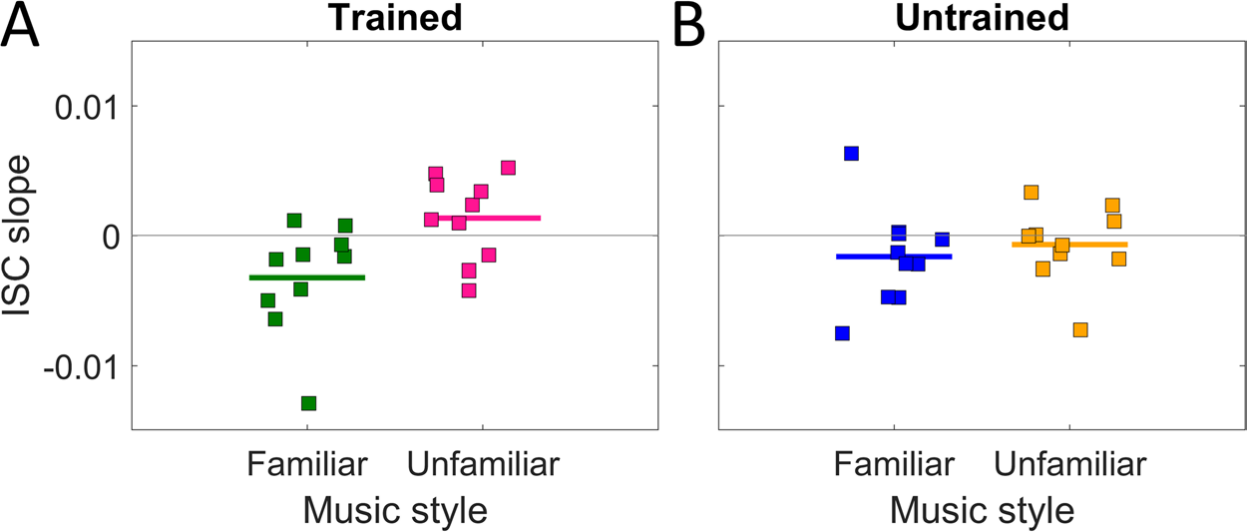
Slope of ISC across repetition per piece differs with musical training. Change in ISC is measured as slope over 3 repeated exposures computed for each of 20 musical pieces (each square is a piece of music). (**A**) Slope is computed for ISC among participants with some musical training. (**B**) Slope computed for ISC among participants who had no training with a musical instrument. Trained participants show a dissociation between familiar and unfamiliar musical styles. Data combines Experiment 2 and 3.

To test the interaction of musical training with repetition and familiarity, while avoiding cumbersome 3-way interactions, we summarize the effect of repetition as the slope of change in ISC across repeats. In our interpretation of ISC as a predictor of engagement, a negative slope would indicate waning engagement across repeated exposures; a positive slope would indicate increasing engagement. The slope of the ISC was computed for each of the 20 musical pieces (Fig. 4) combining Experiment 2 and 3. It seems evident that the slopes differ between familiar and unfamiliar styles for participants with some musical training (Fig. 4A), but not for untrained participants (Fig. 4B). A pairwise t-test confirms this observation (trained: *t*(18) = 2.73, *p* = 1.37e-02, untrained: *t*(18) = 0.62, *p* = 5.43e-01). A post-hoc analysis on the data from Fig. 3 where ISC is measured per subject instead of per music piece further confirms this result. A 2-way repeated measures ANOVA on trained participants alone showed a significant interaction of repeat and familiarity (*F*(1, 67) = 5.65, *p* = 2.03e-02) and a significant interaction for untrained participants (*F*(1, 67) = 4.13, *p* = 4.62e-02). Thus, regardless of whether we analyze effects across participants or across musical pieces, it appears that sustained ISC depends both on familiarity with the musical style as well as musical training of the listener.

### Familiarity and Preference

Whether a particular style is familiar or not may depend on the experiences of each individual participant. In the second and third experiments, we asked each participant to rate their familiarity of the style of each piece on a scale of 1–7. Participant responses conformed to our initial stimulus categorizations of familiar versus unfamiliar music: If we rank pieces by the familiarity score averaged across subjects (see Table 1), we find that the top and bottom half correspond exactly to our predefined judgment of familiar/unfamiliar pieces. We also asked subjects to rate their preference for each piece on a scale of 1–7. Preference and familiarity are highly correlated (r = 0.64). However, we do not find that the level of preference can explain the change in ISC as a function of repeated exposure. When we split the musical pieces in to two groups, high preference and low preference, performing a two-way repeated measure ANOVA, we find a main effect of repetition (*F*(1, 137) = 21.93, *p* = 6.73e-06), but no main effect of preference (*F*(1, 137) = 2.25, *p* = 1.36e-01) and no interaction between repetition and preference (*F*(1, 137) = 1.91, *p* = 1.69e-01).

### ISC during music listening is modulated by attentional state

**Figure 5:**
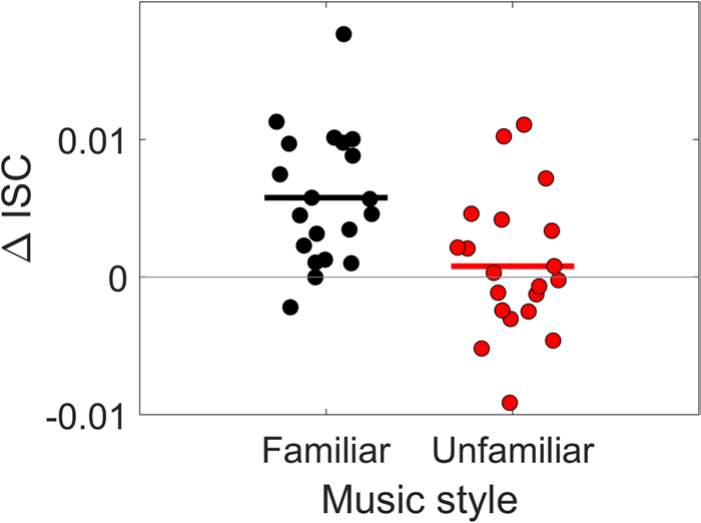
Difference in ISC between attend and distract condition. The ΔISC is computed as the average across musical pieces of the difference between the first repetition and the counting condition (each circle is a participant). There is a larger difference in ISC between attend and distract conditions for familiar music, compared to unfamiliar.

One aspect of engagement is attention to the stimulus. We have previously shown using film stimuli that ISC is modulated by attention^6^, which is consistent with our interpretation of ISC as a marker for engagement. To replicate this finding here with music, participants in Experiment 2 and 3 additionally listened to each musical piece while distracted. In this condition participants were asked to silently count backwards from a random prime number above 800 and below 1000, in steps of 7. We computed the ISC for the first repeat of each 20 musical pieces, for all participants, calling this the *attend condition*. We performed the same computation for the condition in which participants counted backwards, calling this the *distract condition*. The difference between the ISC in the two different conditions can be seen on Fig. 5. These finding were verified by performing a 2-way repeated measure ANOVA, with participants as random effect, and attention and familiarity as the two fixed-effect factors. We find a significant main effect of attention (*F*(1, 57) = 18.46, *p* = 6.83e-05), suggesting that the ISC values found in this experiment relate to attentional engagement and are not only driven by physical properties of the music, e.g. loudness changes. There was also an interaction effect between familiarity and attention (*F*(1, 57) = 10.64, *p* = 1.87e-03). These results suggest that participants were better at ignoring the stimuli they were familiar with, suggesting that it’s harder to disengage when hearing music in an unfamiliar style.

## Discussion

We present evidence that ISC may be capable of tracking musical engagement without behavioral reports, measuring stimulus processing implicitly using EEG. By examining the degree to which participants’ responses match each other, we aim to directly measure how the music “grips” the listener’s brain. When their neural responses proceed in sync, the music is driving their experience.

Because the inter-subject correlation of EEG signals picks up very rapid responses to the music, on the time scale of a second or less, it is unlikely to track explicit cogitation and more likely to track responses that are stimulus-driven. Given that more high-level and explicit responses likely diverge widely from person to person, other methods are needed to assess these aspects of musical responses. Since all participants share exposure to the same time-locked stimulus, synced neural responses likely proceed from the influence of this stimulus, whereas divergent responses likely proceed from more idiosyncratic factors. This method should allow future research to probe the relationship between musical structure and musical engagement, or the influence of various types of extramusical factors on musical engagement. For example, can an intimate performance setting or a pre-concert talk increase musical engagement?

In this study, inter-subject correlation increased when participants listened to music composed in a familiar style compared to music composed in an unfamiliar style. Prior experience with a style shapes listener expectations and provides an entry point for engagement even on the first hearing^18^. Music written in a less familiar style cannot captivate attention as easily or uniformly^19^. Conceptualized in terms of the inverted U-shaped response so prevalent in psychoaesthetics^8^, familiar music can come in closer to the peak on the first hearing, while unfamiliar music can require more exposure to engage listeners. Indeed, as participants listened and relistened to music written in a familiar style, engagement decreased, but this drop did not occur for music written in an unfamiliar style.

Formal musical training modulated these effects. The decrease across repetitions of familiar music was most pronounced for listeners with formal musical training. Their experience with similar music likely made these excerpts sound quite simple to them, precipitating a rapid drop off in engagement with repeated exposures. Unfamiliar music, on the other hand, continued to maintain their attention, offering enough novelty to sustain interest.

Important caveats temper our conclusions. Musical familiarity varies from person to person. We aimed to address this issue by selected musical excerpts based on pilot ratings of style familiarity for each excerpt. Group judgements of familiarity by participants in Experiment 2 and 3 confirmed this categorization for participants in those studies, but individual differences might still have affected results. Additionally, the link between ISC and engagement requires further examination. Although the relationship between ISC and engagement has been thoroughly validated for film^2^, we did not directly test it for music. Yet the present results are consistent with it. For one thing, we replicated the attention modulation (attend/distract) manipulation of Ki, Kelly and Parra^6^ for these musical excerpts. For another, the pattern of ISC changes seems consistent with the predicted trajectory of engagement across repeated exposure. But ultimately the caveat remains, and an appropriate behavioral metric of engagement is needed for validation—potentially listening duration as used in Cohen, Henin and Parra^2^.

The persistence in engagement as measured by ISC across repetitions for unfamiliar style music is unique compared to all other domains for which engagement has been tested using this method. Typically, ISC drops quite consistently across repetitions^6^. Music’s ability under some circumstances to hold attention and engagement across repeats is consistent with theories about some of the domain-specific roles repetition plays in music (Margulis)^5^. We interpret the slope of ISC obtained on trained participants as the persistence of interest in the music. Given this interpretation, listening to the pieces in the experiment in order of the persistence of interest (slope of ISC), suggests that excerpts with a steeper decline in ISC across exposures may have featured more internal repetition and predictable patterning than excerpts that lacked such a decline (see Table 1, which lists the slope of ISC across repetitions with pieces sorted by increasing slope, from decreasing to increasing engagement over repetitions). The excerpt that maintained the most engagement across the three hearings was the one by Thomas Adès, which maintains a loud dynamic level throughout and features the almost consistent introduction of new instruments, timbres, and flourishes as the passage progresses, avoiding the predictability engendered by many of the other excerpts. The excerpt that experienced the steepest drop-off in engagement from first to second hearing was the one by Rossini, which largely features a similarly high volume level, but differs in the iterative use of numerous patterns, rendering the excerpt much more predictable (see Table 1). Novelty in particular may explain the effect of dropping ISC, because novelty is known to drive EEG evoked activity, e.g. P300, mismatch negativity, or error related potentials^18,19^. It may be that as subjects hear the pieces again and again, the surprise effect vanishes, and ISC drops with the diminishing evoked responses. Future research could test this hypothesis more directly with the paradigm presented here.

This paper suggests a new methodology for tracking musical engagement via EEG. It also presents neuroscientific evidence to bolster theories in psychoaesthetics that arose in the 1970s before it was possible to use techniques other than behavioral to investigate them. Future research could harness the potential of measuring ISC to reveal more about how music captivates the mind.
